# Recurrent Sigmoid Volvulus in Pregnancy: Case Report

**DOI:** 10.24248/eahrj.v9i1.818

**Published:** 2025-09-30

**Authors:** Hayte M. Samo, Martini Gemuwang, Fides Canuty, Joshua G. Gidabayda, Emanuel Q. Nuwass

**Affiliations:** a Department of General Surgery, Haydom Lutheran Hospital, Manyara, The United Republic of Tanzania; b Department of Radiology, Haydom Lutheran Hospital, Manyara, The United Republic of Tanzania; c Department of Pediatrics, Haydom Lutheran Hospital, Manyara, The United Republic of Tanzania

## Abstract

Sigmoid volvulus in pregnancy is a rare condition occurring during the third trimester with a high recurrence rate and having fatal complications to both fetus and mother. It requires a prompt diagnosis and multidisciplinary approach.

We present a case of a 33 year old, pregnant woman, Gravida 4, Para 3, at 32 weeks of gestation, who presented with abdominal pain, distension, and tenderness. A Plain abdominal X-ray showed a dilated loop of the sigmoid colon. This was her second admission with similar symptoms. Having previously diagnosed with sigmoid volvulus one month earlier, she was treated conservatively, recovered, and discharged. On this admission she was diagnosed with recurrent sigmoid volvulus, after stabilization she underwent laparotomy which revealed sigmoid colon volvulus at 180 degrees twist. A resection of redundant sigmoid was done, resulting in uneventful postoperative care, discharge, and follow-up. A timely diagnosis and surgical interventions, especially through a multidisciplinary approach during late gestation can lead to favorable outcome for the baby and mother.

## BACKGROUND

Sigmoid volvulus during pregnancy is a rare but serious condition that can lead to significant maternal and fetal complications if not diagnosed and treated promptly. In pregnancy, the enlarging uterus displaces abdominal organs, which may increase the risk of volvulus. Reported incidence of sigmoid volvulus ranges from 1:66,431 to 1:1500 pregnancies.^1^ Most cases occur in the second or third trimester due to increased intra-abdominal pressure and anatomical shifts.^1,2^ Pregnancy can mask classic signs of bowel obstruction, and poses diagnostic and management challenges especially in low-resource settings.

Commonly reported symptoms include abdominal pain, distention, constipation, and vomiting when complicated by peritonitis abdominal tenderness may be present.^3^ The condition being rare requires various modes of treatment according to gestational age.^2^

Timely diagnosis is critical in management of sigmoid volvulus. When diagnosis is delayed beyond 48 hours there is high risk of mortality and morbidity to both the mother and the fetus.^4^ The diagnosis requires high index of suspicion and multidisciplinary team including a general surgeon, obstetrician, pediatrician, and radiologist. Radiological investigation plays a key role as a plain abdominal X-ray may reveal a typical sign such as an inverted U-shaped colonic gas pattern, a coffee bean-shaped appearance and whirl sign. However, fear of radiation exposure to fetus may delay radiologic imaging.^3^

MRI is the preferred radiological investigation due to its safety profile, but CT scan may be considered when there is no MRI.^5^

The treatment depends on gestation age and the presence of peritonitis. At any gestation age, suspected peritonitis and gangrene, warrants emergency explorative laparotomy. In term pregnancy cesarean section may be performed with resection in the same sitting. In early gestation conservative management may be tried with administration of dexamethasone to enhance the fetus lung maturity.^6^

During both the first and second trimester and if there is no sign of peritonitis, enema and rectal tube decompresion can be considered. In the third trimester, sigmoid colectomy is indicated especially if there is peritonitis and gangrene. The non-recurrent sigmoid volvulus with no peritonitis or gangrene may be managed conservatively to promote fetal maturity.^2^ If surgery is indicated in the absence of gangrene, a resection with an end-to-end anastomosis is safe, alternatively in presence of gangrene stoma can be raised and closed in intervals of 2 to 3 months.^6^ There is a need to increase awareness among the healthcare practitioners to enhance their capability on timely diagnosis and intervention of this life-threatening condition.

## CASE PRESENTATION

A 33-year-old pregnant woman, gravida 4, para 3, at 32 weeks gestation age from Iramba district in Singida region, presented with generalized abdominal pain, distension, and constipation for three days with two episodes of vomiting. She was admitted at Haydom Lutheran Hospital in September 2023. During the admission there was no vaginal bleeding or amniotic fluid leakage. She had the same presentation two months prior, and she was managed conservatively, with Nasogastric tube decompression and IV fluids. She improved and discharged a few days later after passing stool and flatus with no abdominal pain.

On the second admission, she had a recurrent painful markedly distended abdomen, and tenderness, making it difficult to assess fundal height. On examination she had stable vitals, with blood pressure of 124/82 mmHg, Heart rate of 71 beats per minute, respiratory rate of 23 cycles per minute, temperature of 37°C, and on abdominal examination, she had a tender grossly distended abdomen, with palpable gravid uterus. Fundal height was estimated to correspond to gestational age of 32 weeks. The Abdomen was hyper tympanic with exaggerated bowel sounds. Rectal examination revealed an empty rectum and no mass. Her laboratory findings revealed stable electrolytes serum potassium 4 mmol/L, blood glucose of 85 mg/dL. Full blood picture with white blood cell count of 6000, Hemoglobin of 14 gm/dL. The provisional diagnosis of acute intestinal obstruction was made by the team. Following the decision of diagnosis, the dexamethasone 4 mg was initiated, however only received two doses as the patient was operated before she finished the four doses due to worsening clinical condition.

Initial management included Nil per oral (NPO), Nasogastric tube decompression, IV fluid, and urethral catheterization. Initial investigations included abdominal Ultrasound complete blood picture and electrolytes panel.

**FIGURE 1:**
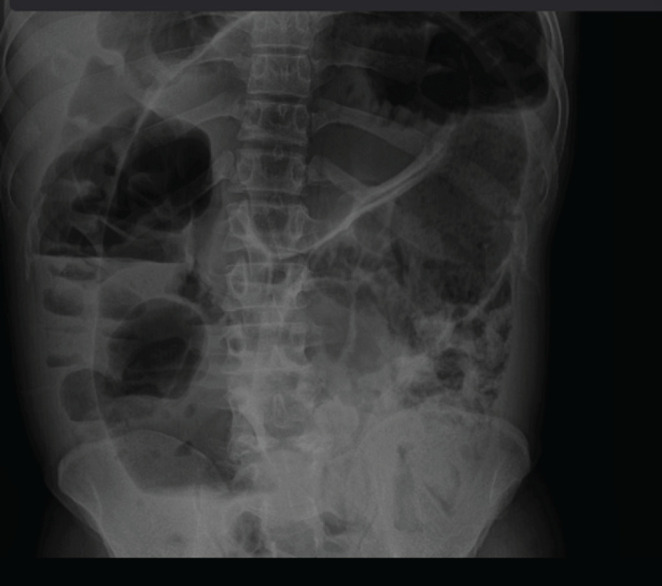
Abdominal Radiograph

Ultrasound revealed a live single intra uterine fetus in a cephalic position with adequate amniotic fluid. Multidisciplinary team including a general surgeon, an obstetrician, and a pediatrician approved a plain abdominal X ray which revealed a bean shaped colonic distention (Figure 1).

This figure demonstrates abdominal radiograph an erect view showing distended colon with coffee bean shaped appearance.

**FIGURE 2:**
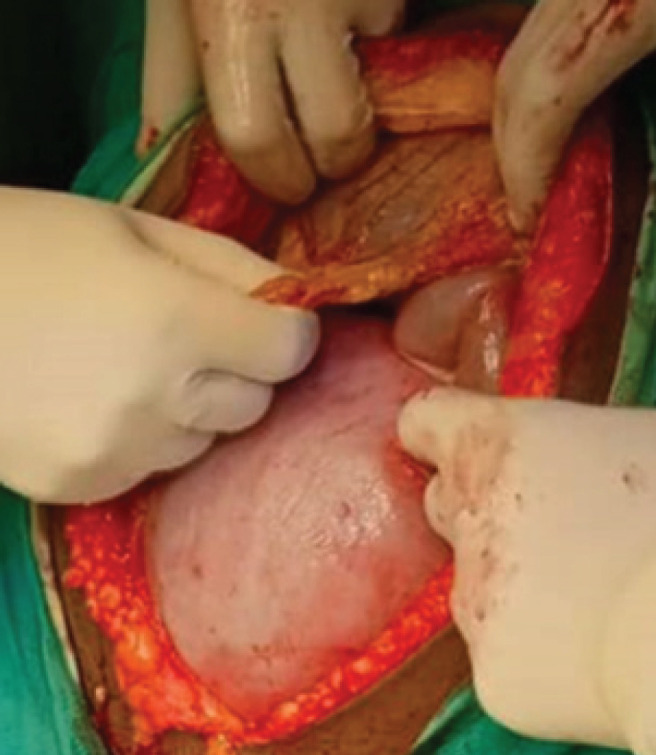
Demonstrates Gravid Uterus just after Midline Incision

**FIGURE 3:**
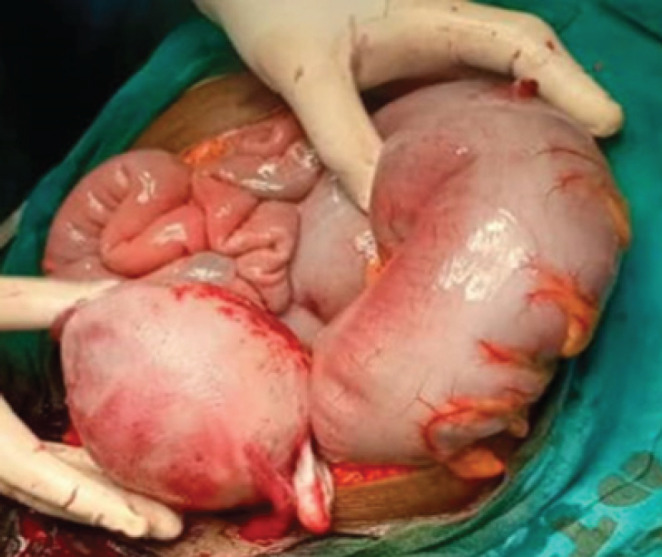
Dilated Loop of Sigmoid Colon

The diagnosis of acute intestinal obstruction was reached, due to recurrent sigmoid volvulus. Saline enema was initiated which was unsuccessful, as abdominal distension and pain persisted and worsened. Emergency exploration laparotomy was planned after counselling and obtaining the consent form for emergency surgery. The patient was prepared for emergency explorative laparotomy which was performed under general anesthesia. A midline incision was performed by a general surgeon assisted by an obstetrician who performed hysterotomy for baby extraction. There was a pediatrician in the operating room to assist in case of fetus prematurity (Figure 2). The intra operation revealed a sigmoid volvulus which was rotated at its axis at 180 degrees with a long and distended viable loop (Figure 3). A hysterotomy was done and a female baby was extracted weighing 1750 Kg. The baby scored 5, 8, and 9 at 1^st^, 2^nd^ and 3^rd^ minutes respectively.

This figure demonstrates dilated loop of sigmoid colon during explorative laparatomy and uterus after hystorotomy matching the clinical and radiological findings of sigmoid volvulus.

The baby was admitted to the neonatal intensive care unit (NICU). The sigmoidectomy was done and end-to-end anastomosis was performed. The patient was admitted to an intensive care unit (ICU) and administered with intravenous fluids, antibiotics and analgesia parenterally for 24 hours then started on ambulation while the baby was admitted in neonatal unit (NICU) for close monitoring and later the mother was discharged to the maternity ward, where she awaited her baby, who was under pediatricians and neonatal nurses care. Three weeks later, the mother and the child who had achieved 1930Kg were discharged home following uneventful post-operative recovery. Follow-up was extended for one year, and during this period, both mother and her child were progressing well.

## DISCUSSION

Recurrent sigmoid volvulus is a very rare cause of intestinal obstruction in pregnancy,^7^ with increased incidence during the third trimester^8^ and it is associated with life-threatening fetal and maternal complications. Pregnancy is a precipitating factor for sigmoid volvulus, as the enlarged gravid uterus in late gestation displaces a redundant or abnormally long sigmoid colon out of the pelvis and twists around its point of fixation where gravid uterus hinders spontaneous untwisting.^8 10^ There are varied clinical presentations of sigmoid volvulus in pregnancy where most of these cases present with abdominal distension, pain, and constipation.^10 11^ This patient presented with the above symptoms and had, in addition, vomiting and abdominal tenderness on deep palpation but without fever.

The diagnosis of sigmoid volvulus in pregnancy at an advanced gestation age is challenging as its presentation can be confused with pregnancy associated complaints.^8^ A high index of suspicion for sigmoid volvulus is required whenever a pregnant woman in her third trimester presents with a triad of abdominal pain, distension, and absolute constipation.^12^ The controversy over imaging options for pregnant mothers pose a diagnostic dilemma, as different literatures recommend that exposure of pregnant mothers to radiation, like X-rays and CT scans of the abdomen, should be avoided.^4^ Meanwhile, other literature reports that MRI is the recommended option, because of its better diagnostic accuracy and safety for the fetus.^6 13 14^ The MRI was the best option for radiological diagnosis for this patient due to absence of radiation risk to fetus however it was not available in our facility. For this case, we had to proceed with a plain abdominal X-ray, which effectively revealed dilatation of the large bowel.

The management approach of sigmoid volvulus in the pregnant patient does not deviate from that of the non-pregnant patient. It involves aggressive resuscitation with intravenous fluid, placement of the nasogastric tube for decompression, and endoscopic reduction of the volvulus. And when this fails, surgery is the definitive option. ^8 15^ Several case reports have shown good results in using endoscopic distortion and rectal tube decompression of sigmoid volvulus during pregnancy where bowel necrosis or vascular occlusion has been ruled out.^16^ In the absence of signs and symptoms of peritonitis, it is reasonable to attempt untwisting and decompression by placement of a soft rectal tube or colonoscopy or sigmoidoscopy.^3,17,18^ The presence of abdominal tenderness and grossly dilated sigmoid colon, in our case, it was a contraindication for the use of endoscopic reduction. The other management option includes a saline enema, which in this case was attempted, but it was unsuccessful, and therefore, the patient proceeded to laparotomy as one of the surgical treatments for cases in which conservative management has failed.^19^

This patient had an extremely dilated sigmoid colon twisted at 180 degrees without gangrene, while in other cases a gangrenous and perforated sigmoid loop were intraoperatively found.^10^ In other cases with peritonitis and perforations, the stoma may be raised and later can be closed after a period of 2 to 3 months.^6,13,14,20^ The anticipated complications include maternal and fetal mortality meanwhile maternal mortality occurs at 5% when the bowel is viable and increases to 50% when there is bowel perforation.^21,22^ The other post-operative complications reported are sepsis and anastomotic leak which are also associated with a high risk of fatal complication to the fetus and the mother.^22^ In our case, caesarean section was performed during laparotomy.

## CONCLUSION

Recurrent sigmoid volvulus in pregnancy is a rare condition that has a high risk of recurrence, and its prompt diagnosis requires a high index of suspicion with the involvement of a multidisciplinary team. The timely diagnosis, management, and surgical intervention are critical following its recurrence at a late gestational age due to favorable outcomes for the baby and mother.

## CONSENT

A written informed consent was voluntarily obtained from the patient for publication of this case report and any accompanying images while maintaining anonymity. A copy of the written and signed consent is available for review.

## References

[1] Ghahremani S, Parisa Razmjouei, Parvaneh Layegh, et al. A Case of Sigmoid Volvulus in Pregnancy: A Rare Emergency in Pregnancy. International journal of pediatrics. 2020;8(1):10743–10747. doi: 10.22038/ijp.2020.45675.3734

[2] Alshawi JS. Recurrent Sigmoid Volvulus in Pregnancy: Report of a Case and Review of the Literature. Diseases of the Colon & Rectum. 2005;48(9):1811–1813. doi: 10.1007/s10350-005-0118-5 15991065

[3] Shaw WZ, Huang CF, Hung TY, Yeh YH. Typical Whirl Sign in Sigmoid Volvulus. ˜The oeJournal of emergency medicine/˜The oeJournal of emergency medicine (Sl Online). 2014;46(3):383–384. doi: 10.1016/j.jemermed.2013.09.017 24412055

[4] Khan MJ, Farid N, Rafique AM, Gul B, Khattak IU. Gangrenous Sigmoid Volvulus in a Complicated Pregnancy: An Alarming Obstetric and Surgical Stigmata. SAJ Case Reports. 2018;5(2):1–5. doi: 10.18875/2375-7043.5.202

[5] Watanabe T, Kinjo T, Yoshino Kinjyo, et al. Sigmoid Volvulus in Pregnancy Assessed by Contrast-Enhanced Computed Tomography Scanning. Case reports in obstetrics and gynecology. 2021;2021:1–4. doi: 10.1155/2021/6692483 PMC7952176 33747587

[6] Tesnière M, Arnoult A, Roger N. Sigmoid Volvulus in Pregnancy. ˜The oeJournal of emergency medicine/˜The oeJournal of emergency medicine (Sl Online). 2018;54(6):e129–e131. doi: 10.1016/j.jemermed.2018.02.038 29681418

[7] Tarik Souiki, Tayeb Ouazzani, Alami B, et al. Sigmoid volvulus in pregnancy. Formosan journal of surgery. 2022;55(6):225–228. doi: 10.4103/fjs.fjs_102_22

[8] Esra DİŞÇİ, Rıfat PEKSÖZ, Kara S, Sabri Selcuk ATAMANALP. Sigmoid volvulus in pregnancy: Current approach in diagnosis and treatment. Journal of surgery and medicine. 2022;6(3):243–245. doi: 10.28982/josam.1072519

[9] Pfeiffer AF, Clark RE, Sullivan J, Syed, Byrne JJ, Boyd AR. Sigmoid volvulus in pregnancy: A rare case report. International journal of gynaecology and obstetrics. 2024;165(3):1285–1287. doi: 10.1002/ijgo.15384 38226725

[10] Lodhia J, Joachim Magoma, Joylene Tendai, Msuya D, Suleiman J, Kondo Chilonga. Sigmoid volvulus in pregnancy: a case report. Journal of medical case reports. 2021;15(1). doi: 10.1186/s13256-021-03151-3 PMC8579578 34753500

[11] Bogale N, Beyene S, Desta D, Tessema E, Sebsbie Birhanie. Cecal Volvulus in Pregnancy, a Diagnostic Dilemma and Management: A Case Report and Literature Review. Open access surgery. 2023;Volume 16: 87–93. doi: 10.2147/oas.s436134

[12] Theresia A. K, Joseph M. L. Sigmoid Volvulus in Second Trimester is Challenge to Obstetric and Surgical Departments at St. Francis Referal Hospital, Kilombero, Tanzania. Clinical Medical Reviews and Case Reports. 2020;7(9). doi: 10.23937/2378-3656/1410319

[13] Ashraf O, Peer S, Fayaz M, Saleem Dar M, Illahi I, Shafi F. Sigmoid volvulus during second trimester of pregnancy in a primigravida: Report of a rare case with review of imaging of sigmoid volvulus. International Journal of Case Reports and Images. 2016;7(7):436. doi: 10.5348/ijcri-201676-cr-10664

[14] Maxim BG, Cimpoca-Raptis BA, Ciobanu AM, et al. Diagnosis and management of intestinal obstruction during pregnancy. Romanian Medical Journal. 2022;69(S2):27–32. doi: 10.37897/rmj.2022.s2.6

[15] Al Maksoud AM, Barsoum AK, Moneer MM. Sigmoid volvulus during pregnancy: A rare non-obstetric complication. Report of a case and review of the literature. International Journal of Surgery Case Reports. 2015;17:61–64. doi: 10.1016/j.ijscr.2015.10.030 26551555 PMC4701819

[16] Atamanalp SS. Endoscopic Decompression of Sigmoid Volvulus: Review of 748 Patients. Journal of Laparoendoscopic & Advanced Surgical Techniques. Published online November 9, 2021. doi: 10.1089/lap.2021.0613 34748412

[17] Aftab Z, Toro A, Abdelaal A, et al. Endoscopic reduction of a volvulus of the sigmoid colon in pregnancy: case report and a comprehensive review of the literature. World journal of emergency surgery. 2014;9(1). doi: 10.1186/1749-7922-9-41 PMC4107753 25057285

[18] Cortez N, Berzosa M, Kiranmayi Muddasani, Kfir Ben-David. Endoscopic Decompression of Recurrent Sigmoid Volvulus in Pregnancy. Journal of Investigative Medicine High Impact Case Reports. 2020;8:232470962097593–232470962097593. doi: 10.1177/2324709620975939 PMC7705807 33238755

[19] Ghodratollah Maddah, Gholam Hossein Kazemzadeh, Abdollahi A, Mostafa Mehrabi Bahar, Tavassoli A, Hossein Shabahang. Management of sigmoid volvulus: options and prognosis. PubMed. 2014;24(1):13–17. 24411535

[20] Ribeiro F, Chechter M, Fábio Piovezan Fonte, et al. Volvulus of the Sigmoid Colon during Pregnancy: A Case Report. 2012;2012:1–5. doi: 10.1155/2012/641093 PMC3335546 22567527

[21] Alrahmani L, Rivington J, Rose CH. Recurrent Volvulus during Pregnancy: Case Report and Review of the Literature. Case Reports in Obstetrics and Gynecology. 2018;2018:1–5. doi: 10.1155/2018/4510754 PMC5852906 29686912

[22] Deniz Simsek, Gulten Ozgen. Recurrent sigmoid volvulus: Cause of colon perforation, sepsis, and fetal death. Journal of obstetrics and gynaecology research. 2021;47(6):2230–2233. doi: 10.1111/jog.14764 33749071

